# EPIK: precise and scalable evolutionary placement with informative *k*-mers

**DOI:** 10.1093/bioinformatics/btad692

**Published:** 2023-11-17

**Authors:** Nikolai Romashchenko, Benjamin Linard, Fabio Pardi, Eric Rivals

**Affiliations:** LIRMM, University of Montpellier, CNRS, Montpellier, France; LIRMM, University of Montpellier, CNRS, Montpellier, France; LIRMM, University of Montpellier, CNRS, Montpellier, France; LIRMM, University of Montpellier, CNRS, Montpellier, France

## Abstract

**Motivation:**

Phylogenetic placement enables phylogenetic analysis of massive collections of newly sequenced DNA, when *de novo* tree inference is too unreliable or inefficient. Assuming that a high-quality reference tree is available, the idea is to seek the correct placement of the new sequences in that tree. Recently, alignment-free approaches to phylogenetic placement have emerged, both to circumvent the need to align the new sequences and to avoid the calculations that typically follow the alignment step. A promising approach is based on the inference of *k*-mers that can be potentially related to the reference sequences, also called phylo-*k*-mers. However, its usage is limited by the time and memory-consuming stage of reference data preprocessing and the large numbers of *k*-mers to consider.

**Results:**

We suggest a filtering method for selecting informative phylo-*k*-mers based on mutual information, which can significantly improve the efficiency of placement, at the cost of a small loss in placement accuracy. This method is implemented in *IPK*, a new tool for computing phylo-*k*-mers that significantly outperforms the software previously available. We also present *EPIK*, a new software for phylogenetic placement, supporting filtered phylo-*k*-mer databases. Our experiments on real-world data show that *EPIK* is the fastest phylogenetic placement tool available, when placing hundreds of thousands and millions of queries while still providing accurate placements.

**Availability and implementation:**

*IPK* and *EPIK* are freely available at https://github.com/phylo42/IPK and https://github.com/phylo42/EPIK. Both are implemented in C++ and Python and supported on Linux and MacOS.

## 1 Introduction

Phylogenetic placement is an increasingly popular task in phylogenetic analysis (Czech *et al.* 2022). Its input is a set of reference sequences (often aligned), a phylogenetic tree representing their evolution, and a (usually large) collection of novel query sequences. The problem is to find, for each query, the likely location(s) of its evolutionary origin in the reference tree. Applications of phylogenetic placement range from taxonomic identification and microbiome analysis (Thompson *et al.* 2017, Janssen *et al.* 2018, Asnicar *et al.* 2020, Bohmann *et al.* 2020), to the inference of new clades (Dunthorn *et al.* 2014, Bass *et al.* 2018), and tracking of viral variants (Singer *et al.* 2020, Turakhia *et al.* 2021).

The interest of phylogenetic placement is particularly evident for amplicon-based analysis of environmental samples. Because of the number and limited length of the sequences typically found in these samples, *de novo* phylogenetic inference would be too unreliable and inefficient on these data (Czech *et al.* 2022). Moreover, phylogenetic placement software plays a key role in an ecosystem of tools for biodiversity quantification and visualization (Czech *et al.* 2020, Barbera *et al.* 2021), sample comparison, and identification of correlations (Czech and Stamatakis 2019).

Early phylogenetic placement tools such as *pplacer* (Matsen *et al.* 2010) and *EPA* [*Evolutionary Placement Algorithm*; Berger *et al.* (2011)] were based on maximum likelihood (ML) inference—a successful approach that provides highly accurate placements. However, those implementations suffered from two scalability problems. First, they were limited by the size of the reference tree, making it problematic to apply them to large phylogenies, e.g. with dozens of thousands of taxa (Koning *et al.* 2021). Second, those methods require to *align each query* to the reference sequences, which is why such methods are called *alignment-based*. Naturally, they also require a multiple alignment of the reference sequences, which is nonetheless often is a prerequisite to estimate the tree (or at least branch lengths and model parameters). The limitation of alignment-based methods is that, even though queries are typically short, their alignment to long reference sequences is poorly scalable with the ever-increasing amounts of sequencing data delivered by current technologies.

Further developments in phylogenetic placement methods focused on overcoming those challenges. *APPLES*, followed by *APPLES-2* (Hasan *et al.* 2020, Balaban *et al.* 2022), was the first method enabling placement to ultra-large phylogenies. It is a distance-based method, where the distances between the query and the reference sequences can be computed on the basis of an alignment, or in an alignment-free way. It allows placing queries to trees of hundreds of thousands of taxa at the cost of lower placement accuracy compared to competing methods. Similar developments include *pplacerDC* (Koning *et al.* 2021) and *SCAMPP* (Wedell *et al.* 2023), which implement heuristics extending the applicability of ML-based placement to large phylogenies.

*EPA-ng—*the successor of *EPA—*significantly improved the ML-based placement speed with massive parallelization (Barbera *et al.* 2019). However, the challenge of aligning query sequences was only addressed by the recent emergence of *alignment-free* methods. *RAPPAS* preprocesses the references by computing *phylo-k-mers* independent of queries (Linard *et al.* 2019). Phylo-*k*-mers are stored for later use during placement. This allows *RAPPAS* to avoid aligning the queries, which explains the “alignment-free” term (even though the input contains aligned reference sequences), and makes it highly scalable in the number of queries to place. Moreover, *RAPPAS* proved to be highly accurate, especially for short queries (Blanke and Morgenstern 2021).

Another recent alignment-free development is *App-SpaM*, a distance-based method that utilizes the concept of spaced words (Blanke and Morgenstern 2021). *App-SpaM* has a number of practical advantages: it is among the fastest phylogenetic placement tools and requires the reference sequences to be neither aligned nor assembled. When reference sequences are aligned and assembled, however, *App-SpaM* is usually not the most accurate tool available (Blanke and Morgenstern 2021).

Here, we take further steps to overcome the challenges of phylogenetic placement and introduce two tools that together form the successor of *RAPPAS*. First, we present *IPK* (*Inference of Phylo-K-mers*), a tool for efficient computation of phylo-*k*-mers. *IPK* improves the running times of the phylo-*k*-mer construction step by up to two orders of magnitude. We also introduce *phylo-k-mer filtering—*a method to reduce large phylo-*k*-mer collections with little or no loss in placement accuracy. These developments improve the scalability of computing phylo-*k*-mers to larger phylogenies. Second, we present *EPIK* (*Evolutionary Placement with Informative K-mers*), an optimized parallel implementation of placement with filtered phylo-*k*-mers. *EPIK* substantially outperforms its predecessor. We provide experiments on placement accuracy and speed, showing that *EPIK* can place millions of short queries on a single thread in a matter of minutes or hours. When placing large collections of queries, *EPIK* outperforms the state-of-the-art in placement speed, while remaining highly accurate.

## 2 Materials and methods

Let *A* denote a *reference sequence alignment*, and *T* denote a *reference phylogenetic tree* whose leaves are in one-to-one correspondence with the sequences of *A*. Sequences can be either composed of nucleotides or amino acids. Besides *A* and *T*, a collection of *query* sequences is also given. Then, for any query *q*, phylogenetic placement aims to identify the branch from which *q* likely diverged from the rest of the tree. Some placement methods also estimate the precise position of the divergence along the identified branch, and the length of the pendant branch terminating in *q*. Note that the reference tree remains fixed, and the queries are not actually added to the tree; this leaves the evolutionary relationships between queries unresolved. Also, we assume that the query sequence is homologous to parts of the reference sequences. This is an essential prerequisite to most phylogenetic placement methods (Czech *et al.* 2022).

**Figure 1.**
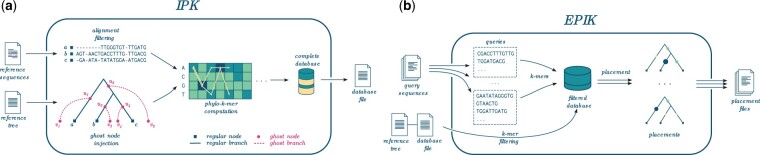
*IPK* and *EPIK*. (a) *IPK* takes an alignment of reference sequences and a reference tree as input. The alignment and tree are preprocessed, most notably the tree is extended by adding ghost nodes and ghost branches. Then state probabilities are computed at every ghost node and alignment site. On the basis of this, a phylo-*k*-mer database is constructed. Finally, mutual information values are computed and stored alongside the rest of the phylo-*k*-mer database for later use. (b) *EPIK* takes the phylo-*k*-mer database (with the corresponding reference tree and mutual information values) and query sequences as input. Depending on the way *EPIK* is called, the whole or a part of the phylo-*k*-mer database is loaded in memory. The *k*-mers of every query are searched in the database to place it onto the reference phylogeny. The result is a phylogenetic placement file (.jplace-formatted), one for each input query file.

As in *RAPPAS*, our method splits phylogenetic placement into two stages (see Fig. 1 for an illustration). First, *IPK* preprocesses *A* and *T* to create a *phylo-k-mer database*. This stage does not require any knowledge of query sequences. Second, *EPIK* uses phylo-*k*-mers to place the query sequences in an alignment-free way, based on the matches between the *k*-mers in the query and the phylo-*k*-mers in the database. Note that once we have the phylo-*k*-mer database computed for a given alignment and tree, we can reuse it to place queries as many times as needed.

### 2.1 Phylo-*k*-mer inference

Phylo-*k*-mers can be thought of as *k*-mers equipped with probabilistic information about their possible branches of origin in the reference phylogeny. More precisely, we define a phylo-*k*-mer for a given *k*-mer *w* and fixed *A*, *T* as a tuple $\left(w,y,S_{y}(w)\right)$, where *y* is a branch of *T*, and $S_{y}(w)$ is a *phylo-k-mer score*. This score is an approximation of the probability of *w* being a substring of an unobserved sequence homologous to those in *A*, that diverged from the rest of *T* somewhere along *y* (Romashchenko 2021).

We compute phylo-*k*-mers in a way that that resembles the one of *RAPPAS* (see Fig. 1a for an illustration). We assume a given stochastic model of sequence evolution, and that the branch lengths of *T* were estimated by maximum likelihood under this model. We also assume that *T* is rooted. First, we filter the alignment by removing the columns that contain a certain user-defined percentage of gaps. Second, we create an extended tree by adding *ghost nodes* and *ghost branches* to *T*: for every branch *y*, we add a node $u_{y}$ at the midpoint of *y*, a new leaf $v_{y}$, and a new branch $\left(u_{y},v_{y}\right)$. Ghost nodes aim to represent hypothetical ancestral sequences, as well as related sequences that diverged in the past but are not directly represented by the references. (See Supplementary Section S6 for some additional arguments on the design of ghost nodes.) We set the length of the new branch to the mean length among all paths from $u_{y}$ to the leaves of *T* that descend from $u_{y}$. This value represents our expectation of how evolutionary distant may be the queries that should be placed onto *y*. Notice that since we average over all descending paths from $u_{y}$, it makes correct rooting an important prerequisite of our method.

Then we use standard techniques for ancestral sequence reconstruction to compute the posterior probabilities of any state (nucleotide for DNA and amino acid for proteins) at any site (position) of the alignment for a given node of the phylogeny. *IPK* can execute *PhyML* or *RAxML-ng* (Guindon *et al.* 2010, Kozlov *et al.* 2019) to perform these computations. This step results in a matrix with as many columns as in *A*, describing posterior state probability distributions for every ghost node *u*. Note that assuming statistical independence of states at different sites of the alignment, this step also defines the probability of any *k*-mer at *u* and any *k* consecutive sites of *A*.

The next step is to find, for any ghost node, all *k*-mers whose probability is greater than a user-defined threshold ε for at least one set of *k* consecutive sites in the alignment. For this step, we apply a new divide-and-conquer algorithm instead of the branch-and-bound algorithm used in *RAPPAS*, because the new algorithm is shown to be faster in practice [see Romashchenko *et al.* (2023) for detail and analysis].

For each *k*-mer *w* generated in the above step, we consider a phylo-*k*-mer $\left(w,y,S_{y}(w)\right)$, where *y* is the branch corresponding to the ghost node where *w* was generated, and $S_{y}(w)$ is set to the maximum probability over all possible choices of *k* consecutive columns in *A*, and over the two ghost nodes corresponding to *y*. Such phylo-*k*-mers can later be retrieved from what we call a *phylo-k-mer database*: a set of *k*-mers mapped to lists of tuples $\left(y,S_{y}(w)\right)$ giving scores of *k*-mer *w* for branches *y* such that $S_{y}(w)>\varepsilon$.

Since such databases can reach huge sizes, and because *k*-mers do not contribute equally to placement inference, we designed *phylo-k-mer filtering*, an approach for selecting informative *k*-mers. For every *k*-mer, we compute a filter value indicating how informative for placement the *k*-mer is, and sort *k*-mers by filter value. We propose two algorithms to form the phylo-*k*-mer database: in memory for speed and on disk for low memory usage. We describe both algorithms in Supplementary Section S2.

### 2.2 Phylo-*k*-mer filtering

One of the main novelties of *IPK* and *EPIK* is the possibility of filtering phylo-*k*-mers. This means selecting the *k*-mers that are the most informative for phylogenetic placement according to a measure derived from information theory. We view phylo-*k*-mer-based phylogenetic placement as a classification problem, where (i) the *observations* to classify are the queries, (ii) the *classes* that they must be assigned to are the branches of the reference tree, and, finally, (iii) the *features* used for the classification of a query are all the possible *k*-mers.

Similarly to feature selection in text classification (McCallum and Nigam 1998), we wish to retain the features (*k*-mers) that maximize the *mutual information* (MI) between the class (branch) variable *Y* and the variable $X_{w}$ indicating whether the *k*-mer *w* is present in the query (i.e. $X_{w}=1$ or 0, depending on whether *w* is present or absent, respectively).

In information theory, the mutual information between these random variables, denoted $I(Y;X_{w})$, is defined as the expected gain of information about *Y* (i.e. reduction in *Y*’s entropy) resulting from observing $X_{w}$ (Cover and Thomas 2006):

$$\begin{array}{c} I(Y;X_{w}):=\;H(Y)-H(Y|X_{w})\\ =P(X_{w}=1)\cdot (H(Y)-H(Y|X_{w}=1))+\\ P(X_{w}=0)\cdot (H(Y)-H(Y|X_{w}=0)) \end{array}$$

Since *EPIK* only uses the presence of *k*-mers (and not their absence) to determine the placement of a query (see Section 2.3), the filtering is based on the following modification of the above formula, which only keeps the term with $X_{w}=1$:

(1) $$MI(w):=P(X_{w}=1)\cdot (H(Y)-H(Y|X_{w}=1))$$

(2) $$=c\cdot S_{w}(\text{log}\;|E(T)|+\sum_{y}\frac{S_{y}(w)}{S_{w}}\;\text{log}\;\frac{S_{y}(w)}{S_{w}}).$$

Here, $S_{w}$ is the total score of *k*-mer *w* [i.e. $S_{w}=\sum_{y}S_{y}(w)$], E(T) is the set of tree branches, and *c* is independent of *w* and thus does not play any role in the filtering of *k*-mers. In Supplementary Section S1, we provide more detail about this formula, including the derivation of (2) from (1) and an alternative explanation of why we only keep the term with $X_{w}=1$ in $I(Y;X_{w})$. Intuitively, selecting *k*-mers maximizing MI(w) favors the ones that both are probable and cause a greater gain of information about the placement branch when observed in the query. A thorough analysis leading to this formula is given by Romashchenko (2021).

We filter phylo-*k*-mers as follows. First, we compute MI(w) for all computed *k*-mers. Second, we sort *k*-mers in descending order of MI(w) and serialize them. We implement this procedure differently for the in-memory and on-disk algorithms; see Supplementary Section S2 for detail. As a result, the database on disk is represented so that more informative *k*-mers are closer to the beginning of the database file. For placement, we deserialize *k*-mers in their order until we reach a certain proportion of the entire database’s size or the memory limit set by the user.

### 2.3 Placement step

On the basis of a precomputed phylo-*k*-mer database, *EPIK* places query sequences in the following fashion (Fig. 1b): First, it loads the entirety or part of the database into memory by applying *k*-mer filtering. This consists in loading *k*-mers with their scores until the given memory limit is reached or the complete database is loaded. At the same time, *EPIK* populates a hashmap from *k*-mers to lists of associated tuples of branches and phylo-*k*-mer scores. Since *k*-mers are stored ordered by MI(w), loading time is linear in the size of loaded information. Then each query is placed independently by searching its constituent *k*-mers in the database. For a given query *q*, a log-likelihood score of placing *q* onto branch *y* is computed as follows:

(3) $$\ell_{y}(q)=\frac{1}{k}\sum_{i=1}^{|q|-k+1}\max\{\;\text{log}\;\varepsilon ,\;\text{log}\;S_{y}(w_{i})\},$$

where $w_{1},w_{2},\ldots ,w_{|q|-k+1}$ are all the *k*-mers in *q*. This formula assumes that the branches that do not appear in any tuple associated to a *k*-mer have a phylo-*k*-mer score ε. Also, note that we intend $\ell_{y}(q)$ to approximate the log-probability of sequence *q* diverging from branch *y*. Therefore, the term 1/k corrects for the fact that the sum above includes the log-probability of most query characters *k* times. For each query, we report a number of top-scored branches together with (approximate) likelihood weight ratios, computed as follows (where *b* is the base of the logarithm):

$$\mathrm{LWR}(q,y)=\frac{b^{\ell_{y}(q)}}{\sum_{x\in E(T)}b^{\ell_{x}(q)}}.$$

### 2.4 Placement accuracy evaluation

To evaluate placement accuracy, we used *PEWO* (*Placement Evaluation Workflows*), a framework recently developed by Linard *et al.* (2021). We evaluated the accuracy of *EPIK*’s placement with and without filtering, as well as the accuracy of the other state-of-the-art phylogenetic placement methods. We used the pruning-based accuracy procedure (*PAC*) of *PEWO* that computes the accuracy of phylogenetic placement as follows. First, given a reference alignment and a reference tree, it randomly selects a subtree to remove (“prune”) from the original tree. Second, it extracts query reads from the sequences associated with the leaves removed. Then, query reads are placed on the remaining tree. Finally, for every placed query read, it computes the *node distance* between the observed placement and the expected placement. Node distance is defined as the number of tree nodes on the path between the branch reported as the query’s top-scoring placement (the observed placement) and the branch that was created as a result of pruning the subtree (the expected placement). We used node distance as it is the most commonly used way of evaluating phylogenetic placement accuracy (Czech *et al.* 2022). Also, *APPLES2* and *App-SpaM* do not output multiple placements along with relative confidence scores and thus do not support evaluating *expected* node distance.

We compared the accuracy of *IPK* and *EPIK* against the latest versions of state-of-the-art phylogenetic placement tools: *pplacer*, *EPA-ng*, *App-SpaM*, *APPLES2*, and *RAPPAS* (see Supplementary Section S5 for details). We used *PhyML* for ancestral reconstruction required by *RAPPAS* and *IPK*. We did not include *EPA* in the evaluation since it is the predecessor of *EPA-ng* and because *EPA-ng* supports *EPA*’s placement algorithm as one of the options. We ran these tools for 50 prunings (prunings and queries are identical for all tools). For each pruning, we report the mean node distance across several 150-bp-long queries generated for that pruning. The number of generated queries per pruning is variable, as it depends on the number of sequences in the pruned subtree, and on the length of those sequences. We ran all tools with their default parameters, except *RAPPAS*, for which we used the defaults of *EPIK*: k=10 for DNA and k=6 for proteins instead of the *RAPPAS*’ default k=8. Generally, using higher values of *k*, we can expect increased placement accuracy; we used these values to facilitate comparison with *EPIK*. We ran *EPA-ng*, *pplacer*, and *APPLES2* using query-reference alignments constructed with *HMMER* in the way it is implemented by default in *PEWO*.

To test the placement accuracy of *EPIK*, we used seven real-world datasets of amino acid and nucleotide sequences, where the number of reference sequences ranged from 140 to 3748. All of these datasets have been previously used as benchmarks for the accuracy of phylogenetic placement methods. Three datasets consist of sequences for widely used metabarcoding markers: *D218*, *D500* (Berger *et al.* 2011), and *D652* (Srinivasan *et al.* 2012, Linard *et al.* 2019). Three others contain full-length viral sequences: *D140* (Berger *et al.* 2011), *HCV* (Linard *et al.* 2019), and *HIV* (Schultz *et al.* 2009). Table 1 gives a short description of these datasets (including two other datasets, *neotrop* and *tara*, that were used to test efficiency).

**Table 1.** Datasets used to evaluate the accuracy or speed of phylogenetic placement.^a^

Dataset	Type	Locus	No. of taxa	**Length (** $\times 10^{3}$ **)**
*D218*	DNA	Bacterial 16S rRNA	218	1.5
*D500*	DNA	Chloroplast rbcL gene	500	1.4
*D652*	DNA	Bacterial 16S rRNA	652	1.3
*neotrop*	DNA	Eukaryote 18S rRNA	512	1.8
*tara*	DNA	Bacterial 16S rRNA	3748	1.4
*HCV*	DNA	Complete genome	155	9.4
*HIV*	DNA	Complete genome	881	9.0
*D140*	AA	Genome-scale	140	1.0

a The “Length” column contains the average length of the sequences, measured in units corresponding to the data type (nucleotides or amino acids).

### 2.5 Placement accuracy with filtering

When we remove phylo-*k*-mers from the database, we expect placement accuracy to decrease. We aimed to answer two questions: how quickly does the accuracy decay with increased filtering, and how much can we filter out without hurting placement accuracy significantly? To investigate this, we performed filtering using a parameter μ∈(0,1] indicating the fraction of the original database to keep (if μ=1, no filtering was performed). That is, if |D| denotes the size of the entire database D, we selected *k*-mers until it was impossible to add the next *k*-mer and its associated tuples, without exceeding the database size of μ⋅|D|.

We used pruning-based accuracy evaluation in the following manner. First, for a given set of input data and a range of values of μ∈(0,1], we computed phylo-*k*-mer databases applying the corresponding filtering ratios μ. Then, we evaluated the mean node distance for 30 prunings for every produced filtered database. We carried out this procedure for both the MI filter and a random filter. The random filter selects *k*-mers in random order until a fraction μ of the original database is kept. As input data, we used the same datasets as in the placement accuracy experiments described in Section 2.4.

### 2.6 Running time and memory consumption

We compare the running time and memory consumption of *IPK* and *EPIK* against other tools using the *RES* workflow of *PEWO* (Linard *et al.* 2021). We used datasets with different tree sizes and alignment lengths: *D652*, *neotrop* (Mahé *et al.* 2017), *tara* (Sunagawa *et al.* 2015), and *HCV*. For *D652* and *tara*, we used ten million of 100–150 bp real-world amplicon reads of bacterial 16S rRNA gene retrieved from the Earth Microbiome Project (Thompson *et al.* 2017, Linard *et al.* 2019). For *HCV*, we used ten million queries of 150 bp simulated for the study of Linard *et al.* (2019). For *neotrop*, we used ten million real-world 18S rRNA reads of various lengths [both the reference and the queries were retrieved from the study of Mahé *et al.* (2017)].

We carried out experiments on a computer equipped with Intel(R) Xeon(R) W-2133 CPU @ 3.60 GHz/8.25 MB Cache/62 GB RAM and an HDD with SATA 3.0 and 64 MB cache. We ran all tools in a single-thread mode (not all of them support parallel execution). For the running time, we consider the total wall clock time; for the RAM consumption, we consider the peak maximum resident set size (RSS). Measurements were averaged over three repeats for every stage of computation of every program.

## 3 Implementation

### 3.1 *IPK*: a standalone tool for computing phylo-*k*-mers

We introduce *IPK*, a new tool for computing phylo-*k*-mers. A few essential differences set it apart from *RAPPAS*’s phylo-*k*-mer computation stage. First, we made *IPK* a standalone tool to ease the development of new applications of phylo-*k*-mers. *IPK* computes a database of phylo-*k*-mers for any given reference *A* and *T* and provides transparent access to this database to query *k*-mers. Applications such as *EPIK* and *SHERPAS* (Scholz *et al.* 2020) can use *IPK* as a black box through the provided API. Second, it applies a new, faster algorithm for phylo-*k*-mer computation (Romashchenko *et al.* 2023). When compared to *RAPPAS* for the default value k=10, *IPK* improves running times by more than two orders of magnitude (see Section 4.4 for experimental results). Third, *IPK* computes mutual information values for phylo-*k*-mers that are later used by *EPIK* for phylo-*k*-mer filtering. By default, *IPK* creates the database in memory and then stores it on disk. Alternatively, it can create the database directly on disk to reduce RAM consumption at the cost of longer execution (see Section 4.4 and Supplementary Section S2). This allows for creating databases of sizes that exceed the amount of RAM available, which was not possible with *RAPPAS*.

### 3.2 *EPIK*: faster phylo-*k*-mer-based phylogenetic placement

We also developed *EPIK*, a new tool for phylogenetic placement with phylo-*k*-mers. It implements phylo-*k*-mer filtering, i.e. can place using only the most informative part of the phylo-*k*-mer database (defined by a proportion of the database size or a certain fixed size in bytes). This allows for placement even if the database is larger than the amount of RAM available. Also, *EPIK* can be run in parallel in shared memory for higher speed (see Supplementary Section S4 for detail and results on parallel performance).

Even when running in a single thread, *EPIK* achieves up to one order of magnitude improvement in placement speed over *RAPPAS* because of more efficient implementation (see Section 4.3 for experimental results). For an example of how to run *IPK* and *EPIK*, see Supplementary Section S7.

## 4 Results

### 4.1 Experiments on placement accuracy

**Figure 2.**
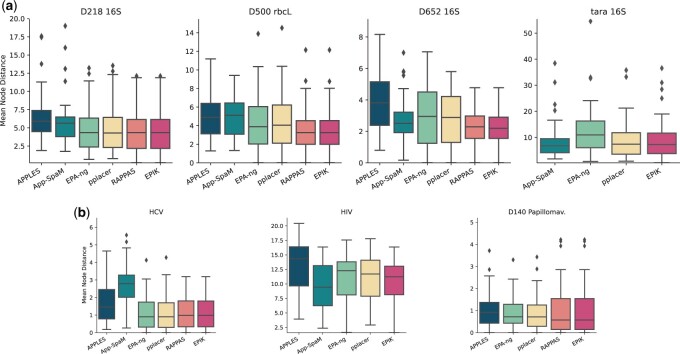
Results on phylogenetic placement accuracy of different tools. One point of the distribution represents placements of multiple queries placed to a particular pruned tree. The *y*-axis corresponds to the mean node distances for such groups of queries and their placements. (a) Metabarcoding markers datasets. (b) Full-genome viral datasets consisting of nucleotide sequences (*HCV*, *HIV*) and amino acid sequences (*D140*).

Here, we assess the placement accuracy of *EPIK* (with unfiltered databases) compared to the state-of-the-art on real-world datasets. Figure 2 shows the distributions of the mean node distance of placements per pruning (lower values are better). For two out of four datasets based on short metabarcoding markers (*D500*, *D652*, see Fig. 2a), *EPIK* shows better accuracy compared to other state-of-the-art tools. These results are to some extent consistent with the ones of Blanke and Morgenstern (2021), where *RAPPAS* using k=8 showed the best accuracy on four out of six 16S datasets.

For the viral datasets (Fig. 2b), *EPIK* was comparable to the best performing methods (*EPA-ng* and *pplacer*) for *HCV* and *D140*, yet was inferior to *App-SpaM* for *HIV*. *App-SpaM* cannot be run for *D140* since it does not support protein sequences. Also, *RAPPAS* could not build phylo-*k*-mer databases for *HIV*, since it exceeded the 32 GB RAM limit.

Figure 2 also shows that, in general, the tools that are based on the same methodology tend to have very similar performance. For example, *EPA-ng* and *pplacer* tend to have very similar node distance distributions; also, the distributions for *RAPPAS* and *EPIK* are matching almost perfectly. However, there exist slight differences between *RAPPAS* and *EPIK* (not visible on Fig. 2). This is due to floating-point rounding that *IPK*+*EPIK* handle better.

Overall, *EPIK* showed similar or better accuracy in six out of seven experiments compared to the state-of-the-art software.

### 4.2 Experiments on filtering performance

**Figure 3.**
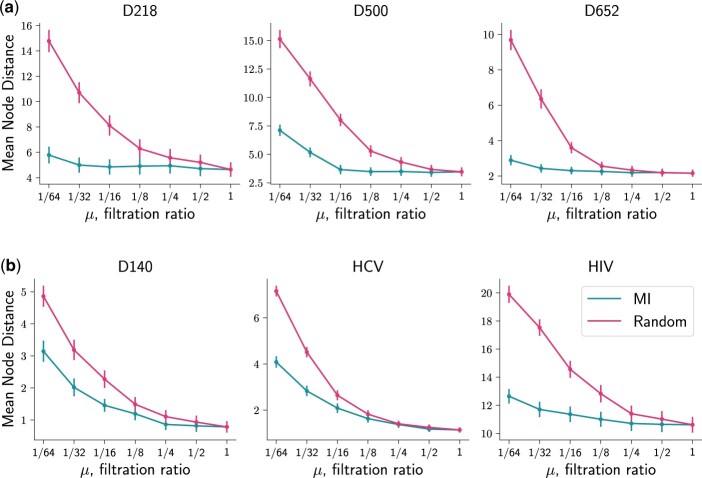
Results on *k*-mer filtering for the mutual information filter and random selection of *k*-mers. The *x*-axis corresponds to the ratio of filtered versus unfiltered database size. The *y*-axis corresponds to the mean node distances of placements (averaged over 30 prunings) obtained with filtered databases. Vertical bars represent standard errors of the means. (a) Results for metabarcoding markers. (b) Results for viral genomes.

Figure 3 shows the placement accuracy results in the experiments with filtered databases. We compare the mean node distance (y-axis) obtained for different degrees of filtration (i.e. different values of μ<1, x-axis) against the mean node distance for μ=1 (complete database) for the same dataset. We also compare the accuracy obtained for two filters—the mutual information (MI) filter and the random filter—obtained with the same value of μ.

First, note that for all datasets and all values of μ<1, MI filtering showed better results than the baseline (always lower node distance than for random filtering). For *D218* (see Fig. 3a), the MI filter decreased the average placement accuracy by 4.4% while keeping only 1/16 of the information compared to the complete database (from 4.65 for μ=1 to 4.85 for μ=1/16). For random filtering, the accuracy loss was 74.8% (from 4.65 to 8.12). For *D500* and μ=1/16, MI filtering reduced the accuracy by 5.7% versus 132% for random filtering. For *D652* and the same filtration ratio, the average accuracy worsened by 6.7% and 66.8% for MI filtering and the baseline, respectively.

For viral datasets (Fig. 3b), *k*-mer filtering had a larger impact on accuracy. For *D140*, keeping μ=1/16 of the database decreased the accuracy by 86% and 190% for MI and the baseline, respectively. For *HCV* and the same μ, the accuracy change was 83% and 132%; still, the drop in accuracy was just 3.2% while halving the database using the MI filter (9.6% for the baseline). For *HIV* and μ=1/16, MI filtering decreased the average accuracy by 7.1% (37.2% for the baseline). Note that results for the *D140* dataset are difficult to compare to those for other viral datasets, because of the different sequence type and length of *k*-mers.

Note that Fig. 3 gives only means and standard errors for every node distance distribution for fixed μ. Supplementary Figure S4 gives a more detailed picture of the distributions. Overall, the results suggest that *k*-mer filtering can significantly reduce the phylo-*k*-mer database size with some (often negligible) decrease in placement accuracy.

### 4.3 Running time: placement

**Figure 4.**
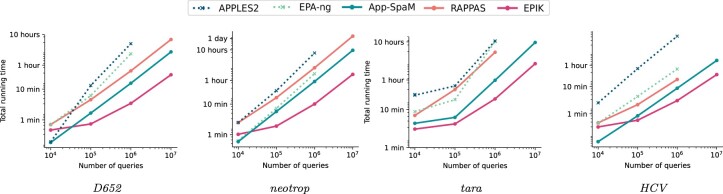
Running time of different phylogenetic placement tools on four reference datasets. Preprocessing time is not counted for alignment-based methods (dotted lines) nor for alignment-free ones (solid lines). Measurements are averaged over three runs.

Here, we evaluate the scalability of the state-of-the-art tools and *EPIK* in the number of query sequences to place. Figure 4 presents measurements of wall-clock time required for placement of increasing numbers of queries. Importantly, the running times do not include any preprocessing needed by these methods. For *EPA-ng* and *APPLES2* by “preprocessing” we mean the alignment of queries to the reference alignment, while for *RAPPAS* and *EPIK* we mean the construction of the phylo-*k*-mer database. *App-SpaM* does not require any preprocessing. We present some results on preprocessing times in the next section. We stopped any jobs that required more than 32 GB of RAM.

*EPIK* showed better placement time than *RAPPAS* in all experiments. The running time improvement over *RAPPAS* varied among datasets and the number of queries placed. For example, while placing ten million queries to *D652*, *EPIK* was around 14 times faster (29 min versus 6 h 49 min); for *neotrop* and ten million queries, it was 18 times faster (1 h 34 min versus 28 h). The lower relative performance of *EPIK* for fewer queries is due to the overhead required by loading the phylo-*k*-mer database in memory that is constant and does not depend on the number of queries placed. We also note that the loaded database size greatly affects the memory consumption of *EPIK* (see Supplementary Table S3 for measurements); this, however, can be reduced with phylo-*k*-mer filtering.

In conclusion, alignment-free approaches showed consistently better running times than alignment-based ones, even if we exclude the time to align query sequences. *EPIK* showed the best running time against all tools on all datasets tested while placing a hundred thousand or more queries.

### 4.4 Running time: phylo-*k*-mer computation

Table 2 gives the averaged measurements of total time spent by *RAPPAS* and *IPK* to compute databases of phylo-*k*-mers for k=10, both using the in-memory and on-disk algorithms. For comparison, it also provides the time needed to align a million queries used in the experiments described in Section 4.3. For all datasets, *IPK* (with the default in-memory algorithm) was more than one hundred times faster than *RAPPAS*: the speed-up was 147×, 124×, 239×, and 120× for *D652*, *neotrop*, *HCV*, and *tara*, respectively. Also, *IPK* clearly outperforms *RAPPAS* in phylo-*k*-mer computation for other *k*-mer lengths (see Supplementary Fig. S5). The speed-up of *IPK* over *RAPPAS* increases with the value of *k*. Importantly, it was significantly faster to preprocess the reference data with *IPK* than to align a million query sequences for all datasets. This was not the case for *RAPPAS*.

**Table 2.** Preprocessing times on four reference datasets.^a^

Dataset	HMMER(1M)	RAPPAS	IPK	IPK (on disk)
*D652*	2 h 21 min	11 h 26 min	4 min 40 s	6 min 46 s
*neotrop*	10 h 00 min	18 h 32 min	8 min 59 s	11 min 23 s
*tara*	2 h 09 min	62 h 59 min	30 min 56 s	46 min 10 s
*HCV*	36 h 58 min	15 h 22 min	3 min 38 s	4 min 45 s

a Preprocessing consists in computing phylo-*k*-mer databases for *RAPPAS* and *IPK*, or aligning query sequences within the reference alignment for alignment-based tools (*EPA-ng*, *APPLES2*) using *HMMER*. For the latter, the running time of aligning one million query reads is shown. Measurements are averaged over three runs.

As for the on-disk algorithm, it showed longer running times than the default in-memory algorithm. Importantly, on-disk processing allowed for a significant reduction in RAM usage. For instance, on-disk *IPK* processed *tara* using 1.6 GB of RAM instead of 30 GB in the in-memory mode, resulting in a database of size 21 GB on disk (see Supplementary Table S2 for additional measurements).

## 5 Discussion

We propose a new two-step solution for alignment-free phylogenetic placement implemented as two modular programs: *IPK* and *EPIK*. First, *IPK* computes a phylo-*k*-mer database from input alignment and phylogeny of reference sequences, then *EPIK* reuses this database to place the query sequences on the phylogeny. The combination *IPK*+*EPIK* follows the strategy used in *RAPPAS* but outperforms it in running time, memory usage, scalability, and introduces significant novelties.

The new algorithm implemented in *IPK* provides dramatic speed improvements: for instance, the *tara* dataset with 3748 species and 1.4 kb alignment, is now preprocessed in minutes instead of days (cf. Table 2). This allowed us to increase the default value of *k* (from k=8 for DNA in *RAPPAS* to k=10), which is a key parameter. Indeed, using longer *k*-mers results in nondecreasing accuracy (Romashchenko 2021). Second, the gain in speed permits to process instances with larger number of species, and hence larger reference trees, which were problematic for *RAPPAS*. Last, *IPK* offers a second computation mode in which the database is computed on disk, allowing users to adapt memory usage to their own resources.

Even if computed on disk, a phylo-*k*-mer database can be large, and thus lead *EPIK* to exhaust memory during placement. However, our filtering experiments show that phylo-*k*-mers are not equally informative for placement. We introduce a novel approach to compute the informativeness of each *k*-mer, and *IPK* sorts the database according to this value. Then *EPIK* can filter the database and load only the most informative part that fits into memory. Accuracy experiments with filtered databases showed that, quite often, a great number of *k*-mers (and associated scores) can be safely excluded. For example, we could reduce the database sizes by a factor of 16 with a minor loss in placement accuracy for datasets of metabarcoding markers, and at least by a factor of 4 for datasets of complete viral genomes.

Let us point out two limitations of *IPK*+*EPIK*. First, they are currently not suited for metagenomic applications that require large reference sequences, like large eukaryotic genomes. For these data, our approach may require using larger values of *k* and limit the possibility of filtering. Hence, extending the applicability of phylo-*k*-mer-based methods to longer genomes seems challenging. Second, the mutual information of a given *k*-mer can only be computed once all its branch-specific scores are available. A perspective for future work is to develop an algorithm to assess *k*-mer informativeness directly during phylo-*k*-mer computation.

Using *EPIK* requires the precomputation of the database with *IPK*, a step that is avoided by alignment-based methods. So how large shall the set of input reads be to counterbalance the precomputation time? In all our experiments (see Section 4.4), aligning a million short reads within the reference sequences was always significantly slower than computing phylo-*k*-mers for the references with *IPK*. Thus, already with circa a million short queries, it is faster to use alignment-free placement with phylo-*k*-mers than alignment-based tools.

Implementing *IPK* as a standalone tool provides flexibility and eases the application of phylo-*k*-mers to other bioinformatic problems, thanks to a carefully designed API. For example, *SHERPAS*, another software based on phylo-*k*-mers, can readily use databases built by *IPK* to infer recombination patterns in viral sequences (Scholz *et al.* 2020). Currently, a tool exploiting phylo-*k*-mers for protein family homology detection is under development. We hope that further phylo-*k*-mer based solutions will be developed in the future.

The C++ implementation of *EPIK* already offers high computational efficiency with a single thread, as well as multi-threading. Currently, for millions of queries or more, *EPIK* is the fastest phylogenetic placement software. Since the combination *IPK*+*EPIK* achieves high accuracy—comparable to state-of-the-art tools—when placing short metabarcoding reads, we hope it will become the tool of choice for large-scale metabarcoding applications.

## Supplementary Material

btad692_Supplementary_Data

## Data Availability

The data underlying this article were obtained from the studies listed in Sections 2.4 and 2.6 and are available from the corresponding sources.
