# Supplementary material for: EPIK: precise and scalable evolutionary placement with informative k-mers
Source: Bioinformatics. 2023 Nov 17;39(12):btad692. doi: 10.1093/bioinformatics/btad692 (PMC10701097; doi:10.1093/bioinformatics/btad692)
Supplement: btad692_Supplementary_Data [file btad692_supplementary_data.pdf]

Supplementary Materials to

EPIK: Precise and scalable evolutionary placement with informative k-mers

Nikolai Romashchenko, Benjamin Linard, Fabio Pardi and Eric Rivals.

November 3, 2023

Contents

S1 Mutual Information-based k-mer filtering **2**

S2 In-memory and on-disk database build **4**

S3 Placement phase **5**

S4 Parallel placement implementation and performance **7**

S5 Placement software versions and parameters **8**

S6 Comparing different ghost node strategies **9**

S7 Workflow example **10**

S8 Supplementary figures and tables **11**

S1 Mutual Information-based k-mer filtering

Recall that Y is the random variable indicating the tree branch where a query should be placed. We assume a uniform prior $\mathbb{P}(Y = y) = 1/|E(T)|$ over all branches of the reference tree. Also recall that X_w is the random variable indicating the presence of k-mer w in the query. Then, $\mathbb{P}(X_w = 1 \mid Y = y)$ is the probability of observing w in a query that diverged from T somewhere along branch y.

To express this probability, we assume a model where the query is generated in a two-stage process as follows. First, a query of the same length as the reference alignment (i.e., $|A|$) is generated as the tip of a lineage splitting off from T at branch y. Second, a substring is sampled from the full-length query, giving the final query q. We let f denote the probability that a k-mer originally present in the full-length query is also present in the final query (which for $|q| \ll |A|$ equals $f = (|q| - k + 1)/(|A| - k + 1)$). We finally assume (as implicitly done in *IPK* and *EPIK*) that the score $S_y(w)$ equals the probability of w being present in the full-length query. We then have:

$$\mathbb{P}(X_w = 1 \mid Y = y) = fS_y(w). \tag{1}$$

Equation 1 is all we need to express the mutual information $I(Y; X_w)$. *In this supplement, we analyse the behavior of* $I(Y; X_w)$*, for* f *in the vicinity of 0.* This corresponds to the case where the query is very short relative to the length of the reference alignment. We will show that, under the model assumptions made above, one obtains the same formula as that given in the main text under Equation 2.

Recall the expression for the mutual information of Y and X_w: [2]

$$\begin{aligned} I(Y; X_w) &= H(Y) - H(Y \mid X_w) \\ &= H(Y) - \mathbb{P}(X_w = 1)H(Y \mid X_w = 1) - \mathbb{P}(X_w = 0)H(Y \mid X_w = 0) \end{aligned} \tag{2}$$

We now express every probability in Equation 2 and in the conditional entropies appearing in it.

$$\begin{aligned} \mathbb{P}(X_w = 1) &= \sum_y \mathbb{P}(X_w = 1 \mid Y = y)\mathbb{P}(Y = y) = \sum_y \frac{fS_y(w)}{|E(T)|} = \frac{fS_w}{|E(T)|} \\ \mathbb{P}(X_w = 0) &= 1 - \mathbb{P}(X_w = 1) = 1 - \frac{fS_w}{|E(T)|} \\ \mathbb{P}(Y = y \mid X_w = 1) &= \frac{\mathbb{P}(X_w = 1 \mid Y = y)\mathbb{P}(Y = y)}{\mathbb{P}(X_w = 1)} = \frac{fS_y(w)}{|E(T)|}\frac{|E(T)|}{fS_w} = \frac{S_y(w)}{S_w} \\ \mathbb{P}(Y = y \mid X_w = 0) &= \frac{\mathbb{P}(X_w = 0 \mid Y = y)\mathbb{P}(Y = y)}{\mathbb{P}(X_w = 0)} = \frac{1 - fS_y(w)}{|E(T)|}\left(1 - \frac{fS_w}{|E(T)|}\right)^{-1} \\ &= \frac{1 - fS_y(w)}{|E(T)| - fS_w} \end{aligned} \tag{3}$$

The conditional entropies in Equation 2 can then be expressed as follows:

$$
\begin{aligned}
H(Y \mid X_w = 1) &= -\sum_y \mathbb{P}(Y = y \mid X_w = 1) \log \mathbb{P}(Y = y \mid X_w = 1) \\
&= -\sum_y \frac{S_y(w)}{S_w} \log \frac{S_y(w)}{S_w} \\
H(Y \mid X_w = 0) &= -\sum_y \mathbb{P}(Y = y \mid X_w = 0) \log \mathbb{P}(Y = y \mid X_w = 0) \\
&= -\sum_y \frac{1 - fS_y(w)}{|E(T)| - fS_w} \log \frac{1 - fS_y(w)}{|E(T)| - fS_w}
\end{aligned}
\tag{4}
$$

We note that all sums above, including the sum that defines S_w as $\sum_y S_y(w)$, are to be intended over all $y \in E(T)$. In practice, since the phylo-k-mer database only stores the scores with $S_y(w) > \varepsilon$, our implementation of filtering assumes $S_y(w) = \varepsilon$ for all missing scores, as done in Section 2.3 in the main text.

Also note that because of the uniform prior on Y, we have $H(Y) = \log |E(T)|$. We now have all the ingredients we need to express $I(Y; X_w)$:

$$
\begin{aligned}
I(Y; X_w) = \log |E(T)| &+ \frac{fS_w}{|E(T)|} \sum_y \frac{S_y(w)}{S_w} \log \frac{S_y(w)}{S_w} \\
&+ \left(1 - \frac{fS_w}{|E(T)|}\right) \sum_y \frac{1 - fS_y(w)}{|E(T)| - fS_w} \log \frac{1 - fS_y(w)}{|E(T)| - fS_w}
\end{aligned}
\tag{5}
$$

Recall that here we are interested in the behavior of $I(Y; X_w)$ in the vicinity of $f = 0$. Application of Taylor's expansion at $f = 0$ allows us to focus on the terms that depend linearly in f:

$$
\begin{aligned}
I(Y; X_w) = \log |E(T)| &+ \frac{fS_w}{|E(T)|} \sum_y \frac{S_y(w)}{S_w} \log \frac{S_y(w)}{S_w} \\
&+ \left(1 - \frac{fS_w}{|E(T)|}\right) \left(\log \frac{1}{|E(T)|} + \mathcal{O}(f^2)\right)
\end{aligned}
\tag{6}
$$

After some simple algebra, this leads to:

$$
I(Y; X_w) = \frac{fS_w}{|E(T)|} \left(\log |E(T)| + \sum_y \frac{S_y(w)}{S_w} \log \frac{S_y(w)}{S_w}\right) + \mathcal{O}(f^2)
$$

Now let $c := f/|E(T)|$. For f sufficiently close to 0, ranking k-mers on the basis of $I(Y; X_w)$ is equivalent to ranking them on the basis of

$$
\begin{aligned}
\frac{1}{c} MI(w) &= S_w \left(\log |E(T)| + \sum_y \frac{S_y(w)}{S_w} \log \frac{S_y(w)}{S_w}\right) \\
&= S_w \Big(H(Y) - H(Y|X_w = 1)\Big).
\end{aligned}
\tag{7}
$$

Note that we have obtained the same formula as that in Equation 2 in the main text. This provides an independent line of reasoning for supporting the use of $MI(w)$ for filtering k-mers. In fact, it is not surprising that the two approaches lead to the same formula: a small

value of f implies that the absence of a k-mer ($X_w = 0$) in the query should provide very little (or no) information gain about the query placement Y. In other words, we can ignore the contribution of $H(Y) - H(Y|X_w = 0)$ to the overall mutual information $I(Y; X_w)$, which is precisely what we did in the main text.

We conclude by noting that selecting k-mers that maximise Equation 7 favors the ones that both are probable (high S_w) and cause a greater information gain about Y if observed in the query (high $H(Y) - H(Y|X_w = 1)$). For more details and experimental work including some alternatives to $MI(w)$, see [5].

S2 In-memory and on-disk database build

This section aims to complete Sec 2.1 of the manuscript by providing more detail about the final stages of creating phylo-k-mer databases. By default, *IPK* creates the database in memory and only then serializes it for later use by *EPIK*. However, if the database is too large and phylo-k-mer filtering is expected, one can create the database externally on disk. This procedure requires substantially less RAM but takes longer to execute. Here we describe both procedures.

On the basis of the rooted reference tree T and the reference alignment A, we extend T by adding ghost nodes and ghost branches as described in the aforementioned section. Then we compute the posterior probabilities of any sequence state at any site of the alignment for every ghost node. We compute phylo-k-mer scores using those probabilities [6]; note that every ghost node — and consequently, every branch — can be processed independently. By processing a branch we mean computing phylo-k-mers for two corresponding ghost nodes and populating a hashmap (*a branch hashmap*) from k-mers to computed scores; we keep the maximum score between two ghost nodes.

Now we come to the point where the on-disk and the in-memory algorithms are different. If the database is built in memory, we merge branch hashmaps into another hashmap (*the database hashmap*) that maps the k-mers to the lists of branch-score pairs. Then we compute Equation 7 for every k-mer and order k-mers by the computed value. Finally, we serialize every entry of the database hashmap in the corresponding order.

Note that the in-memory algorithm keeps the whole database hashmap in RAM during phylo-k-mer and filtering computation. To enable computing databases larger than the amount of RAM available (for potential future filtering), the on-disk algorithm splits k-mers into batches. Precisely, we define a rule that splits the set of k-mers into n_b equal or almost equal non-overlapping batches. In practice, we consider n_b to be a power of two and group k-mers by a shared suffix of a certain length. Once a branch hashmap is constructed, we split it into n_b partial hashmaps. We serialize and unload each of them from memory before processing the next branch.

Once we have processed every branch, we perform filter value computation as follows. For batch i, we deserialize the i-th partial hashmap of every branch and merge them together. This forms a *batch hashmap* that has phylo-k-mers for a certain subset of k-mers. Since the subsets are non-overlapping, we can compute Equation 7 for batch i independently of other batches. We compute mutual information values and serialize the batch again in the order of the computed value. The process is repeated for every batch until we have n_b partial databases of phylo-k-mers (non-overlapping by k-mers contained) on disk that are also sorted by mutual information. Finally, we perform lazy n_b-way on-disk merge of those databases

to create the final database (analogous to the external merge stage of disk-based mergesort). Figure S1 illustrates this process.

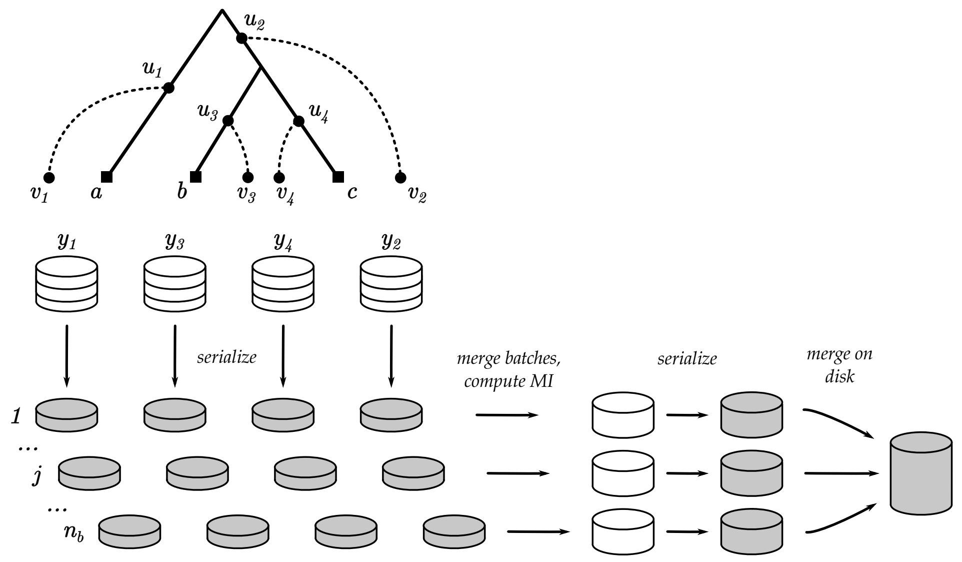

Figure S1: Illustration of the on-disk filtering and merge procedure of *IPK*. Gray and white correspond to storing data on disk and in memory, respectively. Branch hashmaps y_i are split into partial hashmaps and serialized. j-th partial hashmaps of every branch are merged in memory forming the j-th batch hashmap. Then mutual information values for the k-mers in the batch are computed, followed by the sorting of the batch hashmap and its serialization to disk. Finally, the final database is externally merged from sorted batch hashmaps. At any moment, no more than one hashmap among those colored white is kept in memory.

S3 Placement phase

EPIK starts by loading the database from disk applying, if required, k-mer and *score filtering*. By score filtering we mean the possibility to launch *EPIK* with a value of the score threshold ε' different from the original ε that was used to construct the phylo-k-mer database.

Let us consider the memory limit M_{max} and threshold value $\varepsilon' \geq \varepsilon$. *EPIK* computes the maximal number of branch-score pairs that would take no more than M_{max} memory. Then, it deserializes k-mers with the corresponding lists of branch-score pairs and populates the database hashmap excluding tuples with scores under ε'. This process continues until we reach the computed limit. Recall that k-mers are listed in descending order of Mutual Information, meaning that k-mer filtering is automatic.

Afterward, *EPIK* processes the query file in batches. Algorithm 1 describes how one sequence is placed assuming the absence of ambiguous characters in the query. (*EPIK* supports IUPAC codes; in that case it uses a variant of this algorithm; not shown.) For every query, we populate arrays S (branch scores), C (k-mer hit counts), and list L, which tracks branches that were scored.

The first loop scores branches using phylo-k-mers that are found in the database. The second loop accounts for k-mers w that do not explicitly score branch y in the database, in which case we approximate $S_y(w)$ with ε'. The last loop cleans the arrays for the next query's

Algorithm 1: Placement of one sequence without ambiguous characters

```
Data: sequence q, array S, array C, list L
for every k-mer in q do
    Search for the k-mer in the database
    for every tuple (branch y, score s) found do
        if C[y] = 0 then
            Add y to L
        end
        S[y] = S[y] + log s
        C[y] = C[y] + 1
    end
end
for every branch y in L do
    S[y] = (S[y] + log ε' · (|q| − k + 1 − C[y]))/k
end
Select the requested number of branches with highest values of S
Compute likelihood weight ratios
Report selected branches
for every branch y in L do
    S[y] = 0
    C[y] = 0
end
L ← []
```

call. We select the best branches from L and compute their likelihood weight ratios.

We now turn to analyze the time complexity of this algorithm. Let Y_i denote the set of branches that appear associated to the i-th k-mer of the query in the database hashmap. In other words, the branches in Y_i are the ones that are explicitly scored by the i-th k-mer. Note that $|Y_i| \leq |E(T)|$, and before cleaning $L = \bigcup_i Y_i$. Then, for one query, branches are scored in time

$$\mathcal{O}\left(\sum_{i=1}^{|q|-k+1} |Y_i|\right) = \mathcal{O}(|q| \cdot \overline{|Y|}_q),$$

where $\overline{|Y|}_q := \frac{1}{|q|-k+1} \sum_i |Y_i|$ is the average number of branches explicitly scored by k-mers of q.

S4 Parallel placement implementation and performance

We implemented shared-memory parallelism for *EPIK* using OpenMP [3] as follows. *EPIK* reads the database from disk, applying k-mer and score filtering if necessary. It then sequentially reads a batch of queries from disk in a single thread. It places the batch in parallel and outputs the results sequentially. We chose synchronous over asynchronous I/O because it never was the bottleneck in our experiments with various data sizes. Algorithm 1 is applied by every thread to a portion of the batch of queries. Every thread maintains their own arrays S, C, and list L, but they share the read-only database.

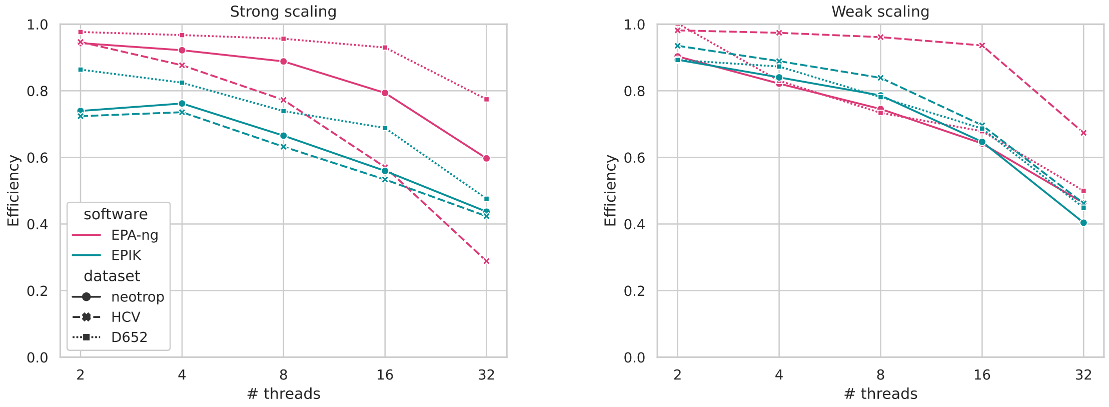

(a) (b)

Figure S2: Scaling of *EPIK* and *EPA-ng* on three datasets. Higher values are better. For *EPIK*, database loading time is not taken into account. Running times considered are the minimums of three runs. (a) Strong scaling. (b) Weak scaling.

Scaling experiments. We conducted two experiments to assess single-node parallel performance of *EPIK* and *EPA-ng* on three datasets: a short marker (*D652*), a complete viral genome (*HCV*), and another short marker (*neotrop*) already used for scaling experiments for *EPA-ng* [1]. In the first experiment, we placed the same set of a hundred thousand queries with different numbers of threads. Let $t_i(n)$ denote the time required to process n queries

using i threads. We computed the strong scaling efficiency for m threads as follows:

$$\frac{t_1(n)}{t_m(n) \cdot m},$$

where $n = 100,000$. In the second experiment, we placed differently sized sets of queries depending on the number of threads and computed the weak scaling efficiency for m threads as:

$$\frac{t_1(n)}{t_m(n \cdot m)},$$

where $n = 10,000$, so that ten thousand queries per thread were placed. Queries placed were subsamples of the ones used for running time experiments (see Section 4.3). Experiments were performed on a machine equipped with Intel(R) Xeon(R) CPU E5-2695 v3 @ 2.30GHz, 35MB cache, and 56 CPUs.

Figure S2 presents the results of these experiments. For *EPA-ng*, results shown are for runs with .fasta-formatted inputs. (We additionally tried to convert the inputs to the .bfast binary format for *EPA-ng* as it claimed to improve its performance; the running times remained almost identical.) *EPIK* demonstrated good-to-moderate strong scaling up to 16 threads (Figure S2a), although it was often inferior to *EPA-ng*. We note that the scaling of *EPA-ng* seems to degrade faster for longer alignments; *EPIK* is less influenced by this. As for weak scaling, *EPIK* again showed good scaling up to 16 threads (Figure S2b).

Additional optimizations. Profiling and experiments on parallel scaling suggest that the placement algorithm of *EPIK* is likely memory and cache bound. Precisely, *EPIK* spends most time performing floating-point operations on the vectors S and waiting for float values to be loaded from RAM. We performed certain optimizations to address this and improved parallel scalability. For better cache locality, we search for all k-mers of the query in the database before updating the arrays S, C, and L with associated branch scores. We implemented phylo-k-mer computation and database serialization in *IPK* so that for every k-mer, associated branch-score pairs are close to be sorted by branch index. This also reduced the number of cache misses while updating the array S. We experimented with memory prefetching of elements S that are about to be updated; this, however, did not lead to any improvement. We implemented updates of S with vectorized instructions (SSE, AVX2, AVX512). This also did not improve running times of placement, likely due to irregular memory layout of such updates.

S5 Placement software versions and parameters

Table S1 gives the versions of the software used for experiments. To align queries within reference alignments, we used `hmmbuild` and `hmmalign` from *HMMER* v.3.3. In all experiments on placement accuracy and time, unless explicitly stated otherwise, we used the default parameters for all programs. The only exception is *RAPPAS*, for which we used non-default lengths of k-mers and disabled skipping alignment sites with gaps during phylo-k-mer computation by setting `--gap-jump-thresh` to 1.0, which corresponds to the default behavior of *EPIK*. We also used *PEWO* for experiments on filtering, placement speed, and accuracy. For that, we modified v1.0.0 by adding the support of *EPIK* to its workflows.

Software	Version
pplacer	v1.1.alpha19-0-g807f6f3
EPA-ng	v0.3.8
App-SpaM	v1.0.3
APPLES	v2.0.11
RAPPAS	v1.21

Table S1: Versions of phylogenetic placement software used in this study.

S6 Comparing different ghost node strategies

Recall that two ghost nodes are added per branch y of the reference tree. One of them (u_y) is added at the midpoint of y, while the other (v_y) is placed at the end of a new branch grafted onto u_y. Here, we call them *inner* and *outer* ghost nodes, respectively.

The inclusion of outer ghost nodes was initially due to the intuition that query sequences can accumulate a number of substitutions *after* their divergence from the reference tree, and therefore they are better modelled by phylo-k-mers originating from both inner and outer ghost nodes [4]. To check the value of this prior intuition, we ran a number of pruning-based accuracy experiments like the ones described in Section 2.4 (setting $k = 8$ for efficiency). In these experiments we compare placement accuracy with databases constructed using both inner and outer ghost nodes (default behavior), to the one with databases constructed using inner ghost nodes only.

The results, shown in Figure S3, tend to confirm that, as expected, with two ghost nodes EPIK exhibits better accuracy on average.

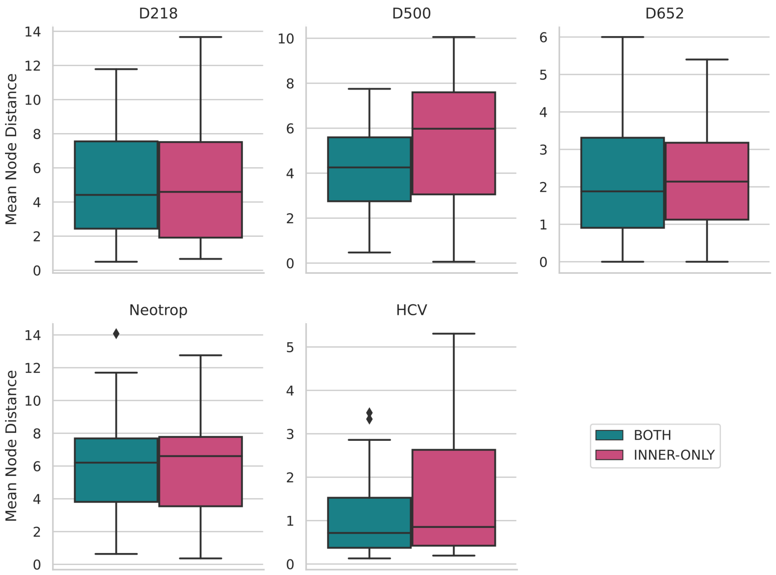

Figure S3: Mean node distance values (PAC procedure, $k = 8$), with different modes for including ghost nodes. In *both* mode: two ghost nodes per branch are used, as described in the main text. In *inner-only* mode: only a single inner ghost node per branch is used.

S7 Workflow example

Here we present examples of commands to run *IPK* and *EPIK*. Assume we have a multiple (DNA) sequence alignment in `reference.fasta` in the aligned FASTA format and a corresponding tree in `tree.newick`. First, we need to run *IPK* to compute a database of phylo-k-mers. This can be done with the following command for $k = 10$:

```
python ipk.py build -s nucl \
                    -r reference.fasta \
                    -t tree.newick \
                    -k 10 \
                    -w output_directory \
                    --ar-config ar.json
```

Note that by default, *IPK* will search for *RAxML-ng* in the environment variables. Also note that we pass the parameters for ancestral reconstruction in a separate config file `ar.json`. Its syntax differs depending on whether we use *PhyML* or *RAxML-ng*. A default version for *RAxML-ng* can look like:

```
{
   "arguments": {
      "data-type": "DNA",
      "model": "GTR+G",
      "opt-branches": "on",
      "opt-model": "on",
      "blopt": "nr_safe"
   }
}
```

IPK will create a database of phylo-k-mers and store it at `output_directory/DB.ipk`. Now one can use this database to place sequences from `query.fasta` on the tree:

```
python epik.py place query.fasta
                     -i output_directory/DB.ipk
                     -o output_directory
                     --max-ram 4GB
                     --threads 8
```

In the end, `output_directory` will contain a `.jplace` file reporting the results of *EPIK*'s phylogenetic placement. See `https://phylo-k-mers.readthedocs.io/` for more details.

S8 Supplementary figures and tables

**Dataset**	**DB size**	***IPK***	***IPK* (on disk)**
D218	2.23	3.73	0.14
D500	2.43	3.90	0.18
D652	3.48	5.41	0.29
neotrop	4.84	6.86	0.29
tara	20.83	30.42	1.55
HCV	1.48	2.44	0.10
HIV	6.13	9.11	0.48

Table S2: **DB size**: file size in GB on disk of complete databases for different datasets (when built with default parameter values). ***IPK*** and ***IPK (on disk)***: peak RAM consumption of in-memory and on-disk *IPK* (using 32 batches) when creating these databases, respectively.

**Dataset**	**APPLES2**	**App-SpaM**	**EPA-ng**	**RAPPAS**	***EPIK***
D652	9.09	1.26	**1.05**	14.61	4.87
neotrop	8.77	2.05	**1.12**	20.79	8.65
tara	5.60	**4.88**	N/A	> 32	23.53
HCV	20.31	**0.95**	1.20	8.12	4.35

Table S3: Peak RAM consumption in GB when placing a million short query sequences for four datasets. Measurements are averaged over three runs.

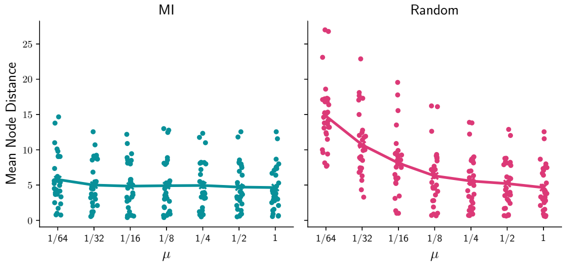

(a) D218

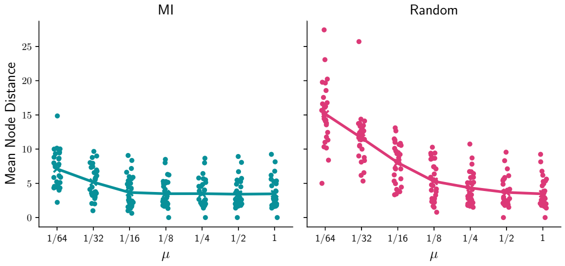

(b) D500

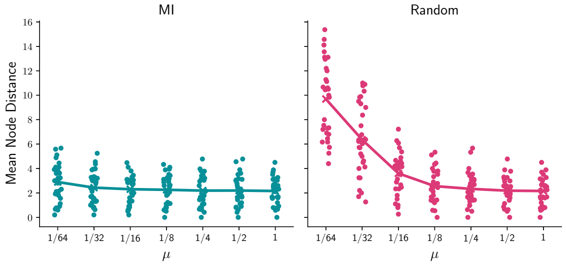

(c) D652

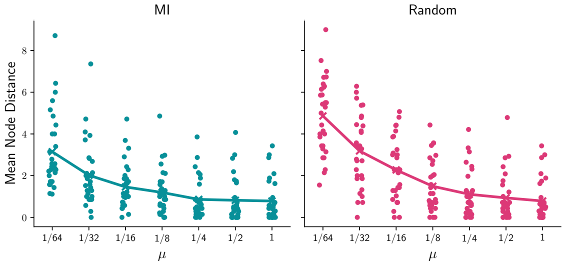

(d) D140

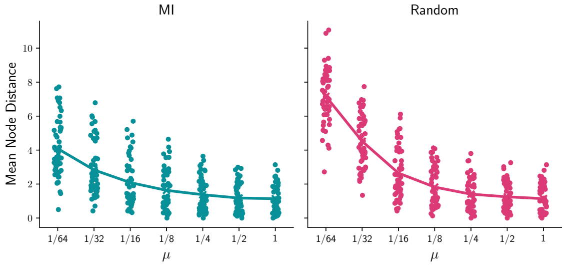

(e) HCV

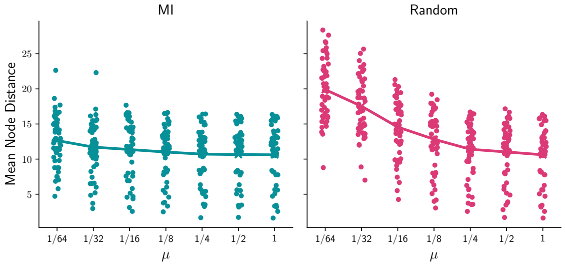

(f) HIV

Figure S4: Mean node distance for each pruning of accuracy experiments with filtered databases on six datasets. This figure represents the same data as in Figure 3 in the main text.

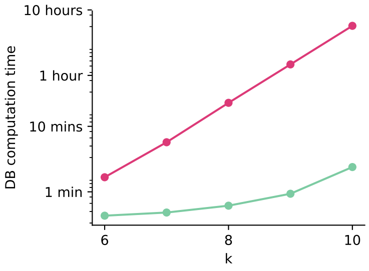

(a) D652

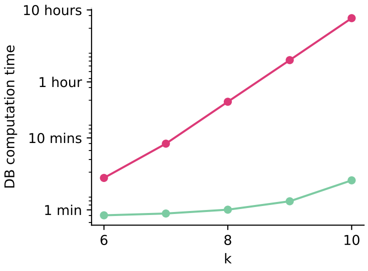

(b) HCV

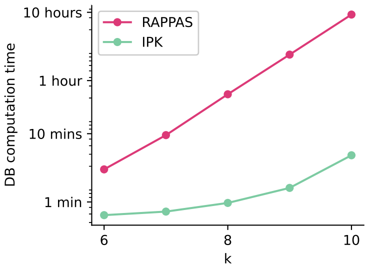

(c) neotrop

Figure S5: Time to compute a database of phylo-k-mers against the size of k for *RAPPAS* and *IPK* on three datasets.

References

[1] Pierre Barbera, Alexey M Kozlov, Lucas Czech, Benoit Morel, Diego Darriba, Tomáš Flouri, and Alexandros Stamatakis. EPA-ng: massively parallel evolutionary placement of genetic sequences. *Systematic Biology*, 68(2):365–369, 2019.

[2] Thomas M Cover and Joy A Thomas. *Elements of Information Theory*. Wiley-Interscience, Hoboken, N.J, 2nd edition, 2006.

[3] Leonardo Dagum and Ramesh Menon. OpenMP: an industry standard API for shared-memory programming. *IEEE computational science and engineering*, 5(1):46–55, 1998.

[4] Benjamin Linard, Krister Swenson, and Fabio Pardi. Rapid alignment-free phylogenetic identification of metagenomic sequences. *Bioinformatics*, 35(18):3303–3312, 2019.

[5] Nikolai Romashchenko. *Computing informative k-mers for phylogenetic placement*. PhD thesis, Université Montpellier, 2021.

[6] Nikolai Romashchenko, Benjamin Linard, Eric Rivals, and Fabio Pardi. Computing phylo-k-mers. *IEEE/ACM Transactions on Computational Biology and Bioinformatics*, 20(5):2889–2897, 2023.
